# Epidemiology of Endometriosis in France: A Large, Nation-Wide Study Based on Hospital Discharge Data

**DOI:** 10.1155/2016/3260952

**Published:** 2016-04-11

**Authors:** Peter von Theobald, Jonathan Cottenet, Silvia Iacobelli, Catherine Quantin

**Affiliations:** ^1^Centre d'Etudes Périnatales de l'Océan Indien (EA 7388), CHU de la Réunion, Site Sud, 97448 Saint-Pierre, France; ^2^Department of Gynecology and Obstetrics, Hospital Felix Guyon, CHU de la Reunion, Site Nord, 9740 Saint Denis, France; ^3^CHRU Dijon, Service de Biostatistique et d'Informatique Médicale (DIM) and Université de Bourgogne Franche-Comté, 21000 Dijon, France; ^4^Néonatologie, Réanimation Néonatale et Pédiatrique, CHU de la Réunion, Site Sud, 97448 Saint-Pierre, France; ^5^INSERM, CIC 1432 and Dijon University Hospital, Clinical Investigation Center, Clinical Epidemiology/Clinical Trials Unit, 21000 Dijon, France; ^6^INSERM UMR 1181 “Biostatistics, Biomathematics, Pharmacoepidemiology and Infectious Diseases” (B2PHI), University Bourgogne Franche-Comté, 21000 Dijon, France

## Abstract

We aimed to assess the prevalence of hospitalization for endometriosis in the general population in France and in each French region and to describe temporal trends, rehospitalization rates, and prevalence of the different types of endometriosis. The analyses were carried out on French hospital discharge data and covered the period 2008–2012 and a population of 14,239,197 women of childbearing age. In this population, the prevalence of hospitalization for endometriosis was 0.9%, ranging from 0.4% to 1.6% between regions. Endometriosis affected 1.5% of hospitalized women of childbearing age, ranging from 1.0% to 2.4% between regions. The number of patients hospitalized for endometriosis significantly increased over the study period (*p* < 0.01). Of these, 4.2% were rehospitalized at least once at one year: ranging from 2.7% to 6.3% between regions. The cumulative rehospitalization rate at 3 years was 6.9%. The types of endometriosis according to the procedures performed were as follows: ovarian (40–50%), peritoneal (20–30%), intestinal (10–20%), and ureteral or bladder (<10%), with significant differences between regions. This is the first detailed epidemiological study of endometriosis in France. Further studies are needed to assess the reasons for the increasing prevalence of endometriosis and for the significant differences in regional prevalence of this disease.

## 1. Introduction

Endometriosis is a frequent illness in young women between 15 and 49 years of age. Its prevalence is estimated at 10 to 50% in the literature [1–3] but no well-conducted nationwide epidemiological studies are available. Its etiology is unknown. Focal ectopic endometrial cells are located mainly in the pelvis, causing intra-abdominal bleeding, inflammation, adhesions, and retractions due to fibrosis. The main symptoms are pelvic pain (dysmenorrhea, dyspareunia, painful defecation or micturation, and painful ovulation) and infertility (due to fallopian tube lesions, adhesions, or direct toxicity of cytokines released by the ectopic cells). Complications such as bowel occlusion, hydroureteronephrosis, ovarian abscess, rectorrhagia, and hematuria may occur. The symptoms are of variable intensity and not always proportional to the extent of the illness. The disease is frequently asymptomatic [3]. The main types of endometriosis are ovarian endometriosis (endometriomas), peritoneal endometriosis, bowel endometriosis, and ureteral and vesical endometriosis. They are frequently found in association depending on the extent of the disease. Less frequently, endometriosis may involve the diaphragm, the pleura, the mediastinum, or the meninges. Diagnosis is difficult and is mainly based on the symptoms, vaginal ultrasound, and MRI imaging, with a delay of 4 to 11 years between the first symptom and diagnosis [4–6]. Diagnosis can only be confirmed by surgery, in most cases by laparoscopy followed by a pathology assessment. Endometriosis is a chronic disease with a recurrence rate of 25 to 50% after conservative treatment [7–9].

As hospitalization as well as surgical investigation is mandatory for the diagnosis, which frequently initiates treatment and involves the most symptomatic patients, we looked at the French hospital discharge database and the Programme of Medicalisation of Information System (PMSI) to assess the prevalence of hospitalization for endometriosis in the population of France. Our main objective was to assess the prevalence of hospitalization for endometriosis in France and in every region of France. Our other objectives were (i) to assess temporal trends in the number of patients, (ii) to assess rehospitalization rates, and (iii) to describe the prevalence of the different types of endometriosis in every French region.

## 2. Materials and Methods

### 2.1. Programme of Medicalisation of Information System

PMSI was established in France in 1991 and extended in 1997 to all French healthcare facilities [10]. It has compiled discharge abstracts for every admission since 2008 and is an instrument for the financial management and each hospital's budget, which depends on the medical activity described in the PMSI. Diagnoses identified during the admission are coded according to the 10th edition of the international classification of diseases (ICD-10).

All procedures performed during the hospitalization are coded according to the French Common Classification of Medical Procedures (CCAM). Each hospital produces its own anonymous standardized data, which are then compiled at the national level. PMSI provides a huge amount of epidemiological information concerning hospitalized French patients [11–19].

### 2.2. Study Design

PMSI abstracts for all patients discharged between 1 January 2008 and 31 December 2012 with a main or associated diagnosis ICD code for endometriosis (N800 to N809) were extracted from the national database. Patients were then separated according to the French region they lived in so as to map the prevalence of endometriosis. Only the first hospitalization was considered and rehospitalizations were analyzed separately. Patients were localized according to their postal code of residence (to assess the prevalence among 15–49-year-old females of each region and the prevalence among 15–49-year-old female patients admitted to hospital).

In all patients with a main diagnosis of endometriosis alone (N800 to N809), we identified the rate of procedures performed during hospitalization or in the following year with the codes of the CCAM. We defined “specific procedures” related to the most frequently involved organs like the peritoneum, bowel, ureter, bladder, and ovaries and quoted the proportion of each specific procedure among all procedures (Table 1).

### 2.3. Statistical Analysis

The Chi square test was used to compare the prevalence of hospitalization among regions and to compare the specific procedure rates with the total procedure rates.

To evaluate trends in the number of women with endometriosis from 2008 to 2012, we used a Poisson regression.

### 2.4. Ethics

This study was approved by the National Committee for Data Protection (registration number 1576793). Written consent was not needed for this study. The data from the PMSI database were transmitted by the national agency for the management of hospitalization data (ATIH number 2015-111111-47-33).

## 3. Results

Hospitalization for a diagnosis (main or associated) of endometriosis occurred in 0.9% of women of childbearing age (between 15 and 49 years of age) in France during the study period. In fact, in a total population of 14,239,197 women of childbearing age in France, 125,178 patients were hospitalized at least once for endometriosis between 2008 and 2012.

**Figure 1:**
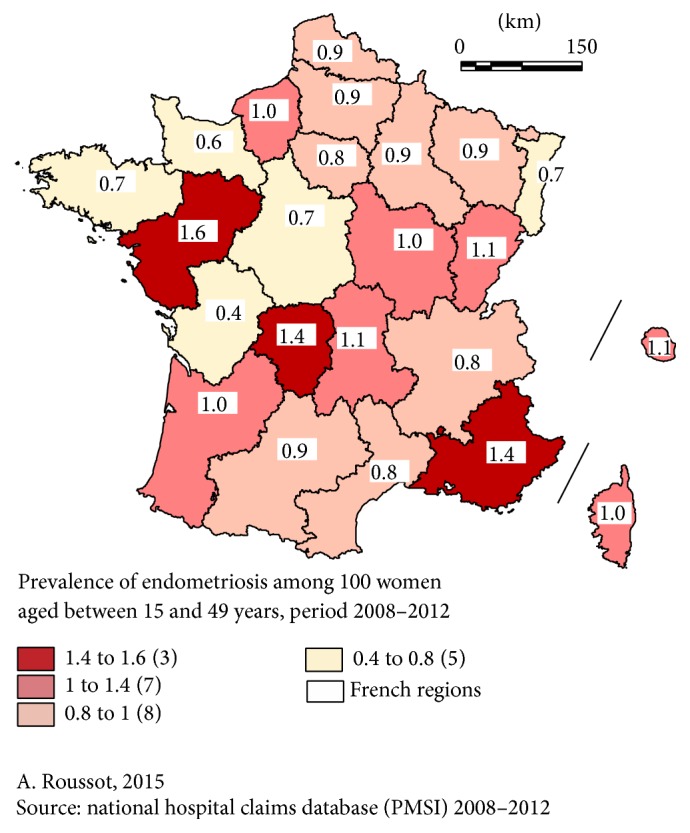
Prevalence of patients hospitalized for main or associated diagnosis of endometriosis in the general population.

The prevalence of hospitalization for endometriosis in the general population according to region ranged from 0.4% (Poitou-Charentes) to 1.6% (Pays de la Loire) (Figure 1).

**Figure 2:**
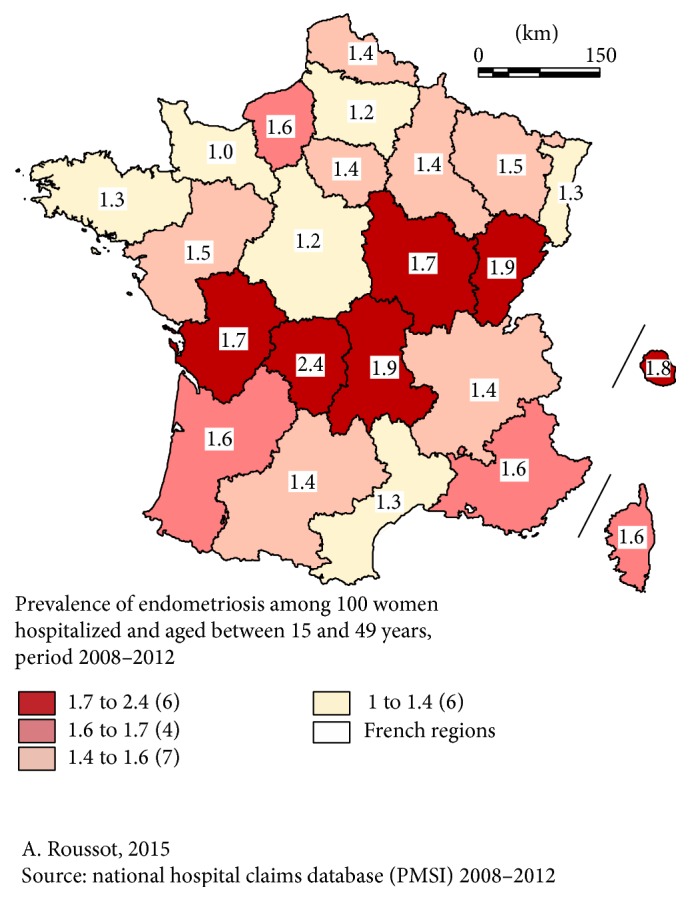
Prevalence of patients hospitalized with a main or associated diagnosis of endometriosis in hospital.

The prevalence in hospital varied in the same way (Figure 2).

Endometriosis was diagnosed in 1.5% of hospitalized female patients between 15 and 49 years of age, ranging from 1.0% (Basse-Normandie) to 2.4% (Limousin). Comparing Figure 1 (prevalence in the general population) and Figure 2 (prevalence in hospital), evident differences appear. For instance, the regions Pays de la Loire and PACA had the highest prevalence in the general population but a lower prevalence in hospital, and the region Poitou-Charentes had the lowest prevalence in the general population but an above-average prevalence in hospital (*p* < 0.01).

The mean age of the 125,178 patients was 37.9 ± 8.0 years.

**Figure 3:**
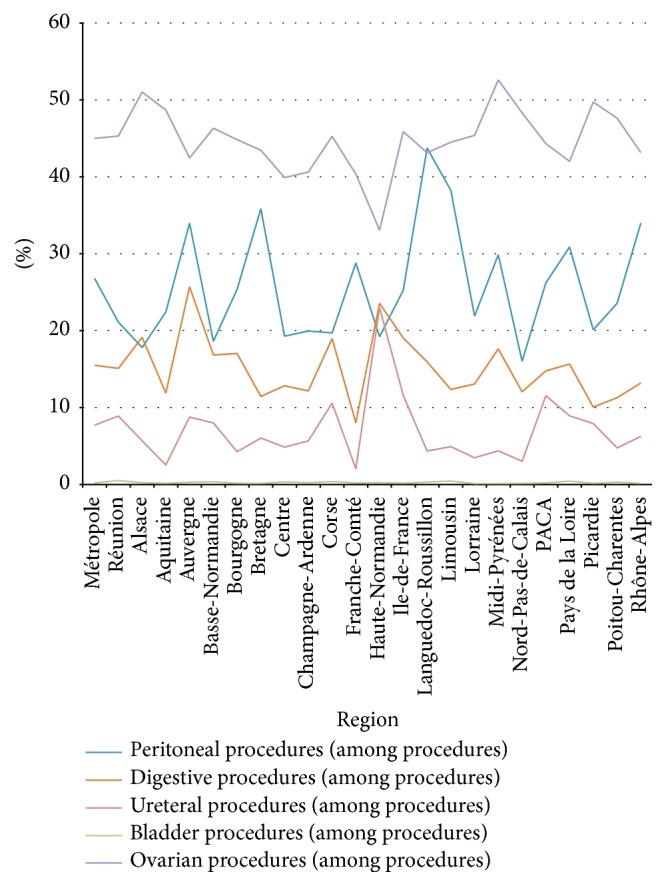
Type of endometriosis according to the procedures performed (% among procedures).

Concerning trends, the number of patients hospitalized for endometriosis increased significantly (*p* < 0.01) from year to year: from 26,492 in 2008 to 28,322 in 2012 (+6.9%), while the population of women between 15 and 49 years of age decreased from 14,455,332 in 2008 to 14,239,197 in 2012. Every year, in France, about 458 more patients were hospitalized for endometriosis although the population of women between 15 and 49 years of age fell by an average of 54,034 per year. Of these patients, 4.2% were rehospitalized at least once at one year during the study period, ranging from 2.7% (region Bretagne) to 6.3% (region Ile-de-France). The cumulative rehospitalization rate in France was 4.1%, 5.6%, and 6.9% after 1, 2, and 3 years, respectively, taking into account the fact that all of the patients during the period 2008–2010 had the necessary follow-up of 3 years. The types of endometriosis according to the procedures performed are shown in Figure 3. There were 40 to 50% of ovarian procedures, mainly removal of endometriomas, 20 to 30% were procedures for peritoneal lesions, 10 to 20% concerned extension of the endometriosis to the bowel, and less than 10% were procedures to cure ureteral or bladder endometriosis. Here again, the rates varied between the regions.

## 4. Discussion

This paper presents the results of the first French study on the epidemiology of endometriosis in a national population-based setting. Few well-conducted studies have reported data on the prevalence of endometriosis and no data are available on its incidence in women without a previous diagnosis [20–22]. Available data consist of prevalence estimates of diagnosed disease among selected hospital or clinical populations (infertile patients, patients with pelvic pain, etc.) and differences in the reported prevalence of the disease vary by as much as 30–40 times [21–23]. For instance, studies that analyzed the frequency of endometriosis in women who underwent surgery for fibroids suggested a prevalence of endometriosis of about 10% [24], but women with fibroids might share the same risk factors as those for endometriosis [25]. Other explanations for these large variations include differences in the indications for surgery, the differing degrees of attention paid by surgeons to the accurate identification of endometriotic lesions, and selective mechanisms that draw patients with suspected endometriosis towards specialized centers [2, 3, 20].

Formal estimates of the prevalence of pelvic endometriosis in the general female population are lacking [21]. In the literature, there is only one rather old study, somewhat similar to our current work. It is based on diagnoses for patients discharged from short-stay nonfederal hospitals in the United States [26]. This study reported that in 1980 endometriosis was a first-listed diagnosis for 97,000 hospital discharges in females between 15 and 44 years of age.

This figure represented 0.9% of all first-listed diagnoses, 1.3% of all first-listed diagnoses minus deliveries, and 6.3% of all first-listed diagnoses of diseases of the genitourinary system (International Classification of Diseases- (ICD-) 9-CM codes 580–629) for females between 15 and 44 years of age. In 1980, women between 15 and 44 years of age in the United States spent an estimated 582,000 days in short-stay nonfederal hospital for health problems for which endometriosis was the first-listed discharge diagnosis. In another study, the annual cost of endometriosis in the United States in 2002 was estimated at $18.8 to $22 billion [23]. In a recent meta-analysis, the overall direct inpatient costs for patients diagnosed with endometriosis were estimated at $12,644 per patient based on the 2002 HCUP database [27]. Our team intends to conduct a further study to estimate the cost of endometriosis in France.

Beyond medicoeconomic studies, it may be interesting to compare the characteristics of regions with very high or very low prevalence in order to identify etiological factors or, at least, risk factors, like the population, pollutants, food habits, or other extrinsic factors. Many of these, mainly pesticide components [28] or other chemicals even in sun lotions [29], have been suspected and assessed in the literature. Of course, genetic factors may also be involved. According to the literature, 30% of endometriosis patients have a family history of endometriosis [30]. Comparison with other countries using the same discharge data system would be interesting as the prevalence in this study already ranges from 0.4% to 1.6% within a single country and as these differences were stable over 5 years.

Our study revealed some marked differences in several regions with regard to prevalence in the general population compared with prevalence in hospital.

These differences were due to other diseases responsible for hospital admissions in the same group of female patients aged 15–49 years, diseases that vary from one region to another. They may also have been caused by the selection of patients: surgery for endometriosis has to be performed in reference centers with a specially trained multidisciplinary team and every region does not have such a surgical department. This means that the two figures give different but complementary information about the prevalence of endometriosis. Our in-hospital prevalence can be compared to the prevalence calculated in the previously cited North American National Center for Health Statistics in 1982 [26]: endometriosis represented 0.9% of all first-listed diagnoses and in our study, 30 years later on another continent, it was 1.5%. Unfortunately, we do not have the numbers in our population in 1982, but we can suppose that the difference could be explained by the increasing trend found in our series and, possibly, by the improvement in healthcare facilities (imaging techniques, laparoscopy, etc.). It may be surprising that, every year from 2008 to 2012, about 458 more patients were hospitalized for endometriosis even though the population of women between 15 and 49 years of age fell by 54,034 per year over the same period in France. As hospitalization concerns only the most symptomatic patients (pain, infertility, and complications), it seems likely that the improvement in imaging techniques is not the main explanation for this significant trend, because asymptomatic or paucisymptomatic patients do not undergo surgery. Either tolerance to symptoms like pain or infertility is decreasing in a society where quality of life is improving (and quality of life is severely impaired by endometriosis [5]) or this illness is really becoming more and more frequent. Our study cannot answer this question but it underlines the notion that if there are more patients in hospital, the cost of endometriosis will rise. Our study shows the relative frequency of the different types of endometriosis resulting in hospitalization in a nonselected population, or more exactly the entire population of a whole country: the most frequent were ovarian and peritoneal endometriosis. No other reports in the literature based on nationwide data collection describe the proportion of operations related to each pelvic organ involved. It might be interesting to compare our findings with those in other countries to see if the relative proportions are the same or if endometriosis has different patterns. There may be different types of endometriosis, possibly related to different etiologic factors in different geographic areas. The rehospitalization rate increased with time: after 3 years, 6 to 7% of women had been back in hospital. Our study cannot say whether this was due to a complication, a recurrence, or another reason like in vitro fertilization, for instance. In the literature, recurrence rates vary between 10 and 56% after 5 years [31, 32] and our rehospitalization rate may appear very low compared with published recurrence rates. However, many recurrent patients do not undergo repeat surgery but are treated medically at home.

There may be some limitations to our study. Given the reliance on ICD-10 codes for the selection of patients and the ascertainment of outcomes, there was a potential for underdetection-related biases for endometriosis even though this disease, when diagnosed, is considered debilitating by gynecologists and thus coded. Coding practices may vary among institutions. Nevertheless, coding quality is checked by medical information professionals in each hospital to correct diagnoses and to increase the recorded comorbidity level. Moreover, as many patients are not admitted to hospital, this study did not assess the prevalence of endometriosis in the whole population but only the prevalence of hospitalization for endometriosis. In fact, the diagnosis of endometriosis is very difficult and frequently delayed [4–6, 23]. This may explain the age of the patients at the time of their hospitalization (almost 38 years). Studies have shown the existence of asymptomatic forms of endometriosis [3, 33–35]. On the other hand, many endometriotic patients suffer from comorbidities, such as adenomyosis, irritable bowel syndrome, and interstitial cystitis, which can all contribute to the symptomatology. The diagnosis of endometriosis can only be confirmed at surgery (usually laparoscopy) as there are no noninvasive diagnostic methods to effectively screen for endometriosis. Pelvic ultrasound and MRI may lead to a suspicion of the disease and sometimes quantify its invasiveness, but these are limited to moderate or severe forms of the disease and are not suitable for the detection of minimal or mild endometriosis. No biomarker has been identified to date [23]. This means that assessing the prevalence of endometriosis in the general population is very difficult because of the complexity of the diagnosis; it may thus be underestimated. The prevalence obtained from the discharge data is interesting because it concerns only the most symptomatic patients, who require hospitalization and thus have surgical proof of endometriosis. These data are robust and are not based on selected categories of patients; they reveal trends in disease prevalence and make it possible to calculate hospital inpatient costs. Another limitation concerns the difficulty of analyzing all of the factors that may explain differences between the French regions. Further research may be needed, including local investigations to collect information that is not available in our data.

## 5. Conclusion

This nationwide study is the first French study to estimate the prevalence of hospitalization for a main or associated diagnosis of endometriosis in each region of the country (0.4% to 1.6%). It revealed a significant trend towards an increase in hospitalizations for endometriosis in France with time. This study also provides information about the relative proportion of procedures for the different types of endometriosis and addresses the question of rehospitalization. This work may be considered the first of many more-detailed epidemiological studies of endometriosis in France in order to study risk factors and to assess the cost of endometriosis in France.

## Figures and Tables

**Table 1:** List of codes for the “organ specific procedures.”

Procedures addressing	CCAM codes		
Peritoneal endometriosis	HPNA001		
	HPNC001		

Bowel endometriosis	JFFA012	HHFA009	HJCA001
	JFFA014	HHFA010	HJCC001
	JFFC001	HHFA011	HJFA002
	HHFA002	HHFA014	HJFA004
	HHFA006	HHFA016	HJFA011
	HHFA008	HHFA017	HJFA017

Ureteral endometriosis	JCCA003		
	JCCC003		
	JCEA001		
	JCEA002		
	JCEA003		
	JCEA005		

Vesical endometriosis	JDFA011		
	JDFA017		
	JDFC023		
	JDCA003		
	JDCC016		

Tubo-ovarian endometriosis	JJFA002	JJFC003	
	JJFA003	JJFC004	
	JJFA004	JJFC006	
	JJFA005	JJFC008	
	JJFA007	JJFC009	
	JJFA008	JJFC010	
	JJFA010		
